# A Cross-Process Signal Integrity Analysis (CPSIA) Method and Design Optimization for Wafer-on-Wafer Stacked DRAM

**DOI:** 10.3390/mi15050557

**Published:** 2024-04-23

**Authors:** Xiping Jiang, Xuerong Jia, Song Wang, Yixin Guo, Fuzhi Guo, Xiaodong Long, Li Geng, Jianguo Yang, Ming Liu

**Affiliations:** 1 Institute of Microelectronics of the Chinese Academy of Sciences, Beijing 100029, China; xiping.jiang@unisemicon.com; 2 School of Integrated Circuits, University of Chinese Academy of Sciences, Beijing 100029, China; yangjianguo@ime.ac.cn; 3 Xi’an UniIC Semiconductors, Xi’an 710075, China; xuerong.jia@unisemicon.com (X.J.); song.wang@unisemicon.com (S.W.); yixin.guo@unisemicon.com (Y.G.); fuzhi.guo@unisemicon.com (F.G.); xiaodong.long@unisemicon.com (X.L.); 4 School of Microelectronics, Xi’an Jiaotong University, Xianning West Road 28#, Xi’an 710049, China; gengli@xjtu.edu.cn

**Keywords:** stacked DRAM, WoW, cross-process analysis methodology, signal integrity

## Abstract

A multi-layer stacked Dynamic Random Access Memory (DRAM) platform is introduced to address the memory wall issue. This platform features high-density vertical interconnects established between DRAM units for high-capacity memory and logic units for computation, utilizing Wafer-on-Wafer (WoW) hybrid bonding and mini Through-Silicon Via (TSV) technologies. This 3DIC architecture includes commercial DRAM, logic, and 3DIC manufacturing processes. Their design documents typically come from different foundries, presenting challenges for signal integrity design and analysis. This paper establishes a lumped circuit based on 3DIC physical structure and calculates all values of the lumped elements in the circuit model with the transmission line model. A Cross-Process Signal Integrity Analysis (CPSIA) method is introduced, which integrates three different manufacturing processes by modeling vertical stacking cells and connecting DRAM and logic netlists in one simulation environment. In combination with the dedicated buffer driving method, the CPSIA method is used to analyze 3DIC impacts. Simulation results show that the timing uncertainty introduced by 3DIC crosstalk ranges from 31 ps to 62 ps. This analysis result explains the stable slight variation in the maximum frequency observed in vertically stacked memory arrays from different DRAM layers in the physical testing results, demonstrating the effectiveness of this CPSIA method.

## 1. Introduction

Today’s computing systems are primarily built on the von Neumann architecture, which reflects a clear separation of processing and memory units [1]. In data processing, a significant amount of data shuttles back and forth between the processing unit and the memory unit, resulting in significant latency and energy costs [2,3,4], forming a critical performance bottleneck [1,4]. The cost of performing a single multiply−accumulate operation by the processing unit is much smaller compared to the cost of moving the associated data [5,6]. The incompatibility between high-density memory processes, such as Dynamic Random Access Memory (DRAM), and logic processes, along with the increasing gap between the performance of memory and processing units, collectively contribute to the memory wall [1,2,3,4,7,8,9,10]. Several near-memory architectures have been proposed to address the memory wall problem by reducing the distance between computation and memory [11,12,13]. In particular, in the near-memory architectures where standard high-density memory and logic process components are integrated into a single package [14,15,16,17,18], cross-process design and analysis methods become a popular research topic [19,20,21].

**Figure 1:**
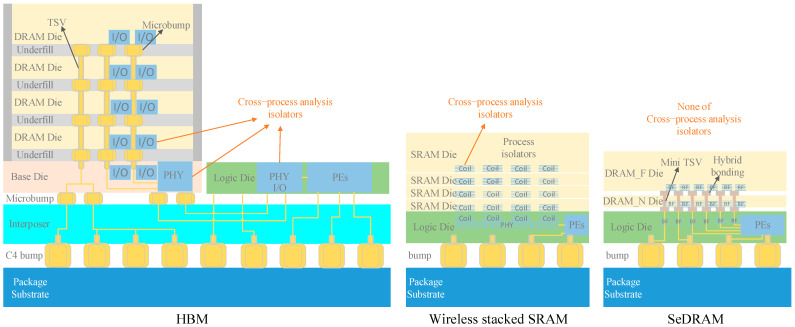
Three near-memory architectures with high-density memory and logic process components integrated into a single package.

In near-memory architectures with high-density memory and logic process components integrated into a single package, cross-process Signal Integrity (SI) design and analysis methods depend on the specific stacking architecture, as shown in Figure 1 and Table 1.

- High Bandwidth Memory (HBM) improves the memory access performance by a Through-Silicon Via (TSV) structured 2.5D near-memory architecture [22,23]. The DRAM dies in HBM are stacked through TSVs and microbumps, forming a DRAM stack. The DRAM stack is horizontally interconnected with logic through an interposer. All DRAM dies and logic dies are independently designed with their respective I/O circuits. These I/O circuits act as isolators for cross-process analysis, segmenting the design and analysis within the HBM package into DRAM, logic, and package-based interconnections. The SI design of constructing an HBM stack is fundamentally packaging design [20].
- Ref. [16] reports a wireless stacked Static Random Access Memory (SRAM) which utilizes semiconductor process coils to establish a vertical data path between four SRAM dies and a logic die, creating a 3D near-memory architecture. In ref. [16], there is no metal-based signal interconnect between SRAM dies and the logic die (“Power supplies are provided via bonded wires”). The interconnection between the two different semiconductor processes is achieved through a magnetic field model within the package of this structure. As a virtual model, the magnetic field model is not constrained by any stacking manufacturing process, simplifying the SI analysis of this structure.

- Refs. [17,18] report a Stacked Embedded DRAM (SeDRAM) architecture, a noteworthy technology in the industry in recent years and the study target of this paper. SeDRAM vertically stacks DRAM dies and a logic die into a hybrid 3DIC package, resulting in the shortest physical distance for memory access at the micron level [24]. Unlike HBM’s packaging integration technology, SeDRAM utilizes a Wafer-on-Wafer (WoW) Back-End-of-the-Line (BEOL) 3DIC process for manufacturing mini-TSV and Hybrid Bonding (HB) to establish high-density vertical memory access interconnects between memory and computing units, significantly enhancing memory access efficiency [25]. In this 3DIC package, a substantial number of mini-TSV and HB cells are used for interconnecting data paths. 3DIC path of SeDRAM is driven by DRAM and logic buffers, creating a cross-process signal integrity analysis environment. As a result, three different semiconductor manufacturing processes, namely, DRAM, logic, and 3DIC, are integrated into the overall design, making it challenging to distinguish boundaries of signal integrity design and analysis. Addressing the aforementioned issues, this paper proposes the Cross-Process Signal Integrity Analysis (CPSIA) method.

In Table 1, among the three near-memory architectures, HBM offers the most convenient simulation framework because the signals across stacks are isolated by I/O. However, HBM has the lowest vertical stacking density. Wireless stacked SRAM achieves an overlapping layout between the coils of the vertical channel and the memory media, achieving an area efficiency of 1162 GB/s/mm^2^ [16], surpassing HBM. The SI analysis of the wireless stacked SRAM structure is conducted on a unit of stacked chips, with interconnections between stacks facilitated by virtual models. SeDRAM, leveraging WoW BEOL, greatly enhances the interconnect density across stacks. However, the I/O-less structure of SeDRAM requires a cross-process SI analysis environment that includes DRAM logic and 3DIC processes.

The SI analysis of HBM and wireless stacked SRAM among the three near-memory architectures listed in Table 1 was conducted on a unit of stacked chips, with system-level simulation implemented between the stacks. The I/O-less structure of SeDRAM requires a cross-process SI analysis environment that includes DRAM logic and 3DIC processes. The signal integrity design and analysis of the SeDRAM architecture presents a significant challenge due to its cross-process nature, encompassing the DRAM, logic, and 3DIC processes. In this hybrid architecture, the memory and computing devices are interconnected through Hybrid Bonding (HB) and mini-TSV cells, with the physical data path across different manufacturing processes in terms of libraries and design rules provided by multiple foundries. Standard Electronics Design Automation (EDA) tools do not support comprehensive SI analysis for these cross-process architectures. To establish sub-micron vertical interconnections between devices of different manufacturing processes, this cross-process vertical interconnection employs buffer drivers for the vertical interconnect units, rather than I/O circuits of HBMs [14,15] or a virtual model of wireless stacked SRAM [16]. Because of the absence of I/O circuits or a virtual model for segmenting the cross-process structure, the SI analysis of SeDRAM is geared towards buffers, essentially following the design requirements of a standard 2D chip. However, this takes place in a 3D cross-process structure. This hybrid architecture demands unique SI design approaches.

This paper addresses the cross-process design and analysis requirements for 3D vertical stacking that are compatible with three different manufacturing processes and proposes the CPSIA method for SeDRAM. This paper formulates lumped circuit models based on the 3DIC physical structure for vertical data paths, facilitating a mixed design and analysis approach that operates independently of 3DIC manufacturing processes. Based on the lumped circuit model, a CPSIA methodology is introduced. It involves the extraction of buffer netlists based on commercial DRAM and logic foundries and the use of the combination of lumped circuits to equivalently represent the vertical stacking paths. A cross-process simulation environment is established, encompassing three commercial processes in terms of DRAM logic and 3DIC. The consistency of the comparative analysis between the simulation results and the silicon results demonstrates the effectiveness of this CPSIA method.

## 2. Study of the 3DIC Model

This section introduces the physical structure of the vertical stacking path used to construct the multi-layer vertical stacked DRAM platform. Following the 3DIC physical structure, a lumped circuit model is proposed, and all values of the lumped elements in the circuit model are calculated with the transmission line model. A 3DIC frequency-domain analysis is demonstrated using the circuit model.

### 2.1. Introduction of Study Target

**Figure 2:**
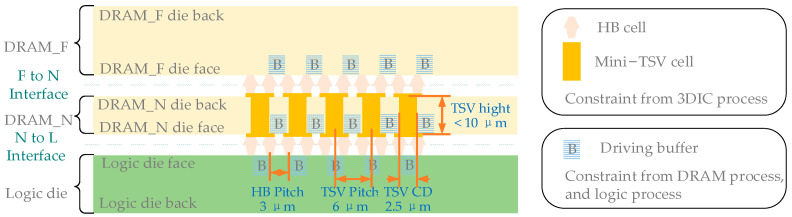
The stacking structure and vertical stacking cells of the SeDRAM.

As shown in Figure 2, the stack of the SeDRAM is the study target of the SI analysis methodology presented in this paper. The DRAM_Near (DRAM_N), DRAM_Far (DRAM_F), and logic components are interconnected through HB and mini-TSV technologies based on the BEOL process, with DRAM_N and DRAM_F representing the DRAM dies located near and far from the logic die, respectively. The HB cell facilitates face-to-back interconnection between DRAM_N and DRAM_F, as well as face-to-face interconnection between DRAM_N and DRAM_F. Mini-TSVs are used to establish interconnections passing through the DRAM_N substrate.

The SI analysis goals for HBM and SeDRAM differ; HBM involves system-level SI analysis, while SeDRAM focuses on cross-process SI analysis. System-level SI analysis is conducted after the completion of chip design. HBM achieves DRAM design based on fixed design targets derived from the system level. It performs system-level SI analysis, including 3DIC, focusing on centralized I/O as the chip-to-chip boundary, resulting in lower analysis precision. In contrast, SeDRAM requires SI analysis for interconnections between different stacks during the design process. It involves chip design optimization based on this SI analysis, necessitating the establishment of a cross-process SI analysis environment that includes DRAM logic and 3DIC processes. The CPSIA method enables higher-precision design, simulation, and optimization during the SeDRAM stacking chip design processes, leading to improved overall system performance.

The physical vertical interconnection is driven by DRAM and logic buffers, and this vertical stacking path involves constraints from the DRAM logic and 3DIC processes separately. The 3DIC is expressed by a lumped circuit model, which helps reduce the complexity of the cross-process simulation environment.

### 2.2. Lumped Circuit Model of Vertical Stacking Paths

**Figure 3:**
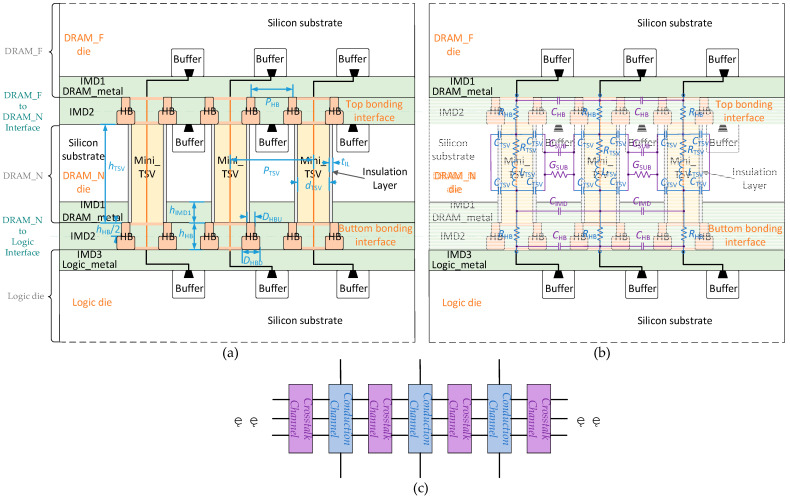
(**a**) Physical structure of vertical stacking paths. (**b**) Lumped circuit model of vertical stacking paths. (**c**) Simplified structures of the conduction channel and crosstalk channel.

Figure 3a presents a physical model of vertical stacking paths. In this model, a 2HB+1TSV+2HB structure is employed to connect memory access signals, which is the primary focus of this work. DRAM_N is interconnected face-to-face with logic, connected by the lower Inter-Metal Dielectric 2 (IMD2) through HB cells. DRAM_F is interconnected back-to-back with DRAM_N, connected by upper IMD2 through HB cells. Mini-TSVs traverse DRAM_N to establish metal connections between the upper and lower HB layers. The circuit in DRAM_F is interconnected with the circuit in logic through the 2HB+TSV+2HB path. This vertical data path is the most complex in SeDRAM and serves as the analysis target because, in SeDRAM, the vertical data paths for the data inputs/outputs (DQs)/command and address inputs (CAs) of the two DRAM dies are individually interconnected with the logic die. For yield and impedance considerations, every two HB cells are interconnected with one mini-TSV, forming the 2HB+1TSV+2HB data path structure.

According to the rule of thumb in transmission line theory, when the length of a transmission line is smaller than 1/20 of the target wavelength (λ), lumped elements can accurately represent the electrical behavior of the transmission line [26]. The target electromagnetic wave of this paper ranges from 1 GHz to 10 GHz, and the wavelength in silicon is from 162,000 µm to 16,200 µm. The lumped model studied in this paper consists of transmission lines in the sub-10 µm range, which is much smaller than the wavelength of the target frequency, satisfying the 1/20 λ condition. Therefore, lumped elements are used to model the vertical stacking cells.

The size of vertical stacking cells is in the sub-10 µm range, as shown in Table 2, and their electrical behaviors can be approximated using lumped elements, as illustrated in Figure 3b. Lumped elements can be categorized into two groups. The first corresponds to the lumped elements resulting from the vertical stacking cells themselves, marked in blue, while the second corresponds to the lumped elements resulting from interactions between vertical stacking cells, marked in purple. The blue lumped elements form a conduction channel, and the purple lumped elements form a crosstalk channel. The simplified structures of the conduction channel and crosstalk channel are depicted in Figure 3c.

In Figure 3b, the blue lumped elements primarily represent the signal path between DRAM_F and logic dies, running from top to bottom, and include the following:

- $R_{\mathrm{HB}}$, the equivalent resistance of the dual HB cell structure;
- $R_{\mathrm{TSV}}$ and $L_{\mathrm{TSV}}$, the equivalent resistance and inductance of the mini-TSV cell connecting the backside and top metal layers of DRAM_N;
- $C_{\mathrm{TSV}}$, the distributed capacitance formed by the outer surface of the mini-TSV copper pillar and the DRAM_N substrate, enclosed by the insulation layer (SiO2) surrounding the TSV.

The purple lumped elements in Figure 3b arise from the medium between vertical stacking cells and manifest in two types of structures. The IMD2 is formed by the BEOL process to create HB cells, exhibiting good insulating properties but possessing a significant relative dielectric constant (see Table 3). Due to the thinning of the DRAM_N substrate, the DRAM_N substrate in the structure of Figure 3a consists solely of the p-type substrate, which has both non-ideal conductivity and a significant relative dielectric constant (see Table 3). Distributed parameters exist in the IMD2 and DRAM_N substrate media, serving as coupling channels for crosstalk between adjacent vertical stacking paths, and their equivalent lumped elements are as follows:

- $C_{\mathrm{HB}}$, the distributed capacitance formed by the adjacent dual HB cell structures through the IMD2 medium;
- $C_{\mathrm{IMD}}$, the distributed capacitance formed by the adjacent mini-TSV cells through the Inter-Metal Dielectric 1 (IMD1) medium (the metal layer of DRAM_N);
- $C_{\mathrm{Sub}}$ and $G_{\mathrm{Sub}}$, the equivalent capacitance and conductance formed by the adjacent mini-TSV cells through the medium of the DRAM_N substrate.

The lumped elements in the lumped circuit model can be determined using the formulas of the transmission line model, including coaxial line, two-wire line, and planar line models [27]. The calculation of these lumped elements is dependent on physical dimensions and material parameters, which are detailed in Table 2 and Table 3, respectively.

$R_{\mathrm{TSV}}$ is calculated using the cylindrical resistor formula:

(1) $$R_{\mathrm{TSV}}=\rho_{\mathrm{TSV}}\times \frac{h_{\mathrm{TSV}}}{\pi \times {\left(d_{\mathrm{TSV}}/2\right)}^{2}}\;\;.$$

$L_{\mathrm{TSV}}$ is determined through the coaxial cable model formula:

(2) $$L_{\mathrm{TSV}}=\frac{\mu_{0}\times \mu_{r\_\mathrm{TSV}}}{2\pi }\times \ln\left(\frac{P_{\mathrm{TSV}}}{\left(d_{\mathrm{TSV}}/2\right)}\right)\times h_{\mathrm{TSV}}\;\;.$$

$R_{\mathrm{HB}}$ represents the parallel resistance of two HB cells, where the resistance of each HB cell is computed in two parts based on the HB structure and using the cylindrical resistor formula:

(3) $$R_{\mathrm{HB}}=\frac{1}{2}\left(R_{\mathrm{HBU}}+R_{\mathrm{HBD}}\right)=\frac{1}{2}\left(\rho_{\mathrm{HB}}\times \frac{h_{\mathrm{HB}}/2}{{\pi \times \left(d_{\mathrm{HBU}}/2\right)}^{2}}+\rho_{\mathrm{HB}}\times \frac{h_{\mathrm{HB}}/2}{\pi \times {\left(d_{\mathrm{HBD}}/2\right)}^{2}}\right).$$

$C_{\mathrm{TSV}}$ represents the distributed capacitance of the mini-TSV in the insulation layer, and it is calculated using the coaxial line capacitance formula:

(4) $$C_{\mathrm{TSV}}=\frac{1}{4}\left(\frac{2\pi \times \varepsilon_{0}\times \varepsilon_{r\_\mathrm{IL}}}{\ln\left(\frac{\left(d_{\mathrm{TSV}}/2+t_{\mathrm{IL}}\right)}{\left(d_{\mathrm{TSV}}/2\right)}\right)}\times \left(h_{\mathrm{TSV}}-h_{\mathrm{IMD}1}\right)\right)\;.$$

$C_{\mathrm{HB}}$ denotes the distributed capacitance of the dual copper pillar structure in IMD2 and is calculated in two parts using the two-wire line capacitance formula:

(5) $$C_{\mathrm{HB}}=C_{\mathrm{HBU}}+C_{\mathrm{HBD}}=\frac{\pi \times \varepsilon_{0}\times \varepsilon_{r\_\mathrm{IMD}}}{{cosh}^{-1}\left(\frac{P_{\mathrm{HB}}}{d_{\mathrm{HBU}}}\right)}\times \frac{h_{\mathrm{HB}}}{2}+\frac{\pi \times \varepsilon_{0}\times \varepsilon_{r\_\mathrm{IMD}}}{{cosh}^{-1}\left(\frac{P_{\mathrm{HB}}}{d_{\mathrm{HBD}}}\right)}\times \frac{h_{\mathrm{HB}}}{2}\;.$$

$C_{\mathrm{IMD}}$ represents the distributed capacitance of adjacent mini-TSVs in IMD1, calculated using the two-wire line capacitance formula:

(6) $$C_{\mathrm{IMD}}=\frac{\pi \times \varepsilon_{0}\times \varepsilon_{r\_\mathrm{IMD}}}{{cosh}^{-1}\left(\frac{P_{\mathrm{TSV}}}{d_{\mathrm{TSV}}}\right)}\times h_{\mathrm{IMD}1}.$$

$G_{\mathrm{Sub}}$ is the distributed conductance of adjacent mini-TSVs in the DRAM_N substrate, determined by the two-wire line capacitance formula:

(7) $$G_{\mathrm{Sub}}=\frac{\pi \times \sigma_{\mathrm{Sub}}}{{cosh}^{-1}\left(\frac{P_{\mathrm{TSV}}}{d_{\mathrm{TSV}}}\right)}\times \left(h_{\mathrm{TSV}}-h_{\mathrm{IMD}1}\right)\;.$$

$C_{\mathrm{Sub}}$ represents the equivalent capacitance of adjacent mini-TSVs in the DRAM_N substrate and is calculated using the two-wire line capacitance formula:

(8) $$C_{\mathrm{Sub}}=\frac{\pi \times \varepsilon_{0}\times \varepsilon_{r\_\mathrm{Sub}}}{{cosh}^{-1}\left(\frac{P_{\mathrm{TSV}}}{d_{\mathrm{TSV}}}\right)}\times \left(h_{\mathrm{TSV}}-h_{\mathrm{IMD}1}\right)\;.$$

The values of all lumped elements in Figure 3b are determined using the method described above, as summarized in Table 4. Among these elements, $R_{\mathrm{TSV}}$, $L_{\mathrm{TSV}}$, $R_{\mathrm{HB}}$, and $C_{\mathrm{TSV}}$ impact conduction along the signal path, whereas $C_{\mathrm{HB}}$, $C_{\mathrm{IMD}}$, $G_{\mathrm{Sub}}$, and $C_{\mathrm{Sub}}$ constitute coupling channels for crosstalk between adjacent vertical stacking paths.

The lumped elements associated with conduction functionality primarily consist of, approximately, a 100 mΩ resistor and a 10 fF capacitor, affecting the data channels, which are comparable to the distributed parameters of metal layers within 2D chips. The lumped elements related to crosstalk functionality primarily consist of, approximately, a 0.1 mS conductance and a 1.5 fF capacitance, establishing crosstalk between data channels, which is comparable to the distributed parameters of metal layers within 2D chips. The scale of distributed numerical values introduced by 3DIC is not fundamentally different from 2D chip designs. Therefore, SeDRAM employs buffers to drive the vertical stacking paths and operates at the DRAM core frequency. The driving units and speeds of 3DIC are the essential differences between SeDRAM and HBM. It is necessary to conduct cross-process signal integrity analysis in combination with SeDRAM’s dedicated driving methods.

### 2.3. Frequency-Domain Analysis

**Figure 4:**
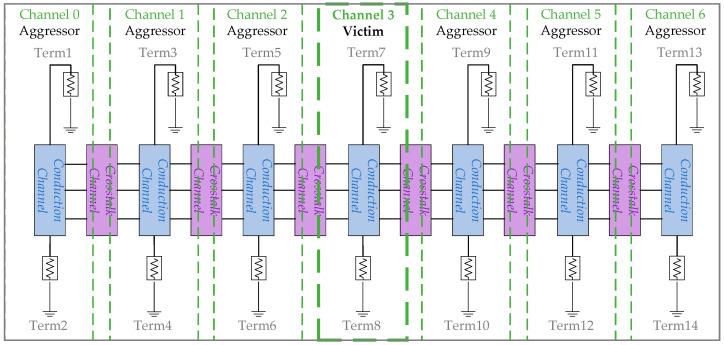
Frequency-domain analysis frame.

The framework for frequency-domain analysis introduces seven channels of lumped circuits for vertical stacking paths, aiming to analyze the channel characteristics of vertical stacking paths, including 3DIC crosstalk, as illustrated in Figure 4. Channel 3 in the middle is considered the victim, while the three outer pairs of channels act as the aggressors. Fourteen terminations are distributed on both sides of the seven channels for S-parameter analysis. Each channel incorporates components such as $R_{\mathrm{TSV}}$, $L_{\mathrm{TSV}}$, $R_{\mathrm{HB}}$, and $C_{\mathrm{TSV}}$ for conduction, as well as $C_{\mathrm{HB}}$, $C_{\mathrm{IMD}}$, $G_{\mathrm{Sub}}$, and $C_{\mathrm{Sub}}$ for crosstalk. In this setup, channel 3 near the center of the framework is chosen as the subject of analysis.

**Figure 5:**
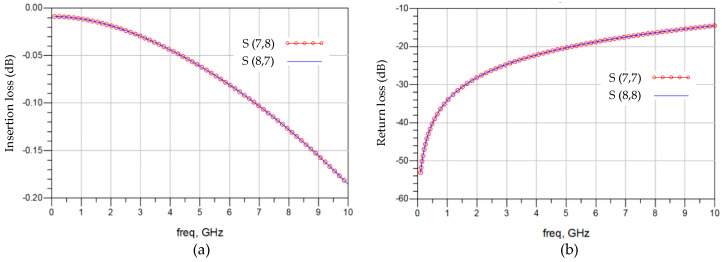
Frequency-domain analysis. (**a**) Insertion loss of channel 3. (**b**) Return loss of channel 3.

Figure 5 illustrates the frequency-domain analysis for channel 3. In Figure 5a, the insertion loss on channel 3 is displayed in terms of the frequency response ratio between termination 7 to 8 and termination 8 to 7. Insertion loss represents the proportion of signal loss from the input to the output caused by the 3DIC path, with values closer to zero indicating better performance. The insertion loss on channel 3 is greater than −0.2 dB below 10 GHz, indicating that insertion loss is not the primary factor affecting 3DIC signal integrity. The return loss on channel 3 is depicted in Figure 5b, including the frequency response ratio between termination 7 to 7 and termination 8 to 8. Return loss characterizes the proportion of the input signal reflected back to the input terminal through the 3DIC path compared to the input signal. Typically, a value lower than −30 dB does not significantly affect channel performance. In SeDRAM, vertical stacking paths operate at the DRAM core frequency, which is lower than 1 GHz, resulting in a return loss below −34 dB, which has no significant impact on signal integrity. However, above 2.5 GHz, the return loss exceeds −25 dB, becoming a major challenge for signal integrity.

**Figure 6:**
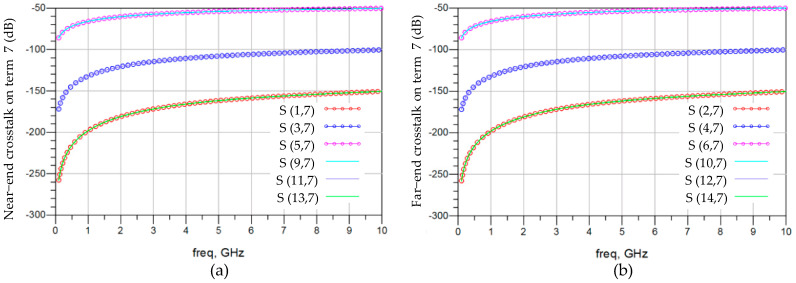
Frequency-domain analysis for the impacts of crosstalk on channel 3. (**a**) Impacts of near-end crosstalk on termination 7. (**b**) Impacts of far-end crosstalk on termination 7.

Figure 6a,b demonstrate the impacts of near-end crosstalk and far-end crosstalk on channel 3. Taking Figure 6a as an example, six frequency response ratios are overlaid, representing the near-end crosstalk impacts of the six aggressors from terminations 1, 3, 5, 9, 11, and 13. Terminations 5 and 9 are closest to termination 7 and have the greatest crosstalk impacts on termination 7, with an impact of −75 dB at 1 GHz. The farther the near-end terminations are from relative termination 7, the less their impact on termination 7. The patterns and numerical values of far-end crosstalk on channel 3 in Figure 6b are similar to near-end crosstalk. Therefore, crosstalk is not a significant challenge to signal integrity.

**Figure 7:**
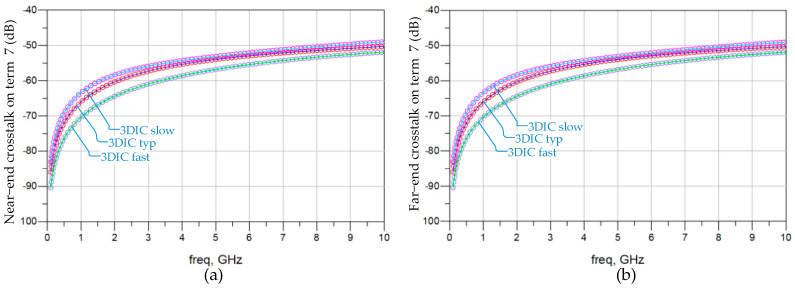
Frequency-domain analysis for the crosstalk on channel 3 with the impact of 3DIC lumped element variation. (**a**) Near-end crosstalk on termination 7. (**b**) Far-end crosstalk on termination 7.

Figure 7 demonstrates the crosstalk on channel 3 with the impact of 3DIC lumped element variation, considering the statistical variations of the vertical stacking path. Under the 3DIC slow condition, all lumped element values of the vertical stacking path are increased by 40%, corresponding to the lowest slew rate of digital signals. Conversely, the 3DIC fast condition involves reducing all lumped element values by 40%, reflecting the highest slew rate. Among the near-end crosstalk response and the far-end crosstalk response, the 3DIC fast condition introduces the least crosstalk. Both the 3DIC fast and slow conditions introduce crosstalk response deviations of less than 6% on the basis of −65 dB at 1 GHz.

This section, in conjunction with the stacking structure and the vertical stacking cell features of the vertical stacked DRAM platform, highlights the distinct nature of WoW SI analysis: cross-process and the absence of process segmentation by I/O circuit. The lumped circuit based on the 2HB+1TSV+2HB structure is introduced to establish a modeling methodology for the vertical stacked DRAM platform. All values of the lumped elements in the circuit model are calculated with reference to the transmission line model. Frequency-domain analysis of vertical stacking paths based on lumped circuits is presented, highlighting that the impact of 3DIC channels increases with the frequency, and the influence of 3DIC channels below 1 GHz meets the design requirements of SeDRAM.

## 3. Cross-Process Analysis

To address the buffer driving (I/O less) method, a cross-process timing-domain analysis method is established, where the 3DIC is represented in the form of a lumped circuit; the DRAM and logic buffers are represented in netlist form, and they are integrated into one simulation environment. Employing this method, an impact analysis introduced by 3DIC crosstalk is demonstrated, coupled with memory access behavior across the 3DIC.

### 3.1. CPSIA Method

In the SeDRAM architecture, the vertical stacking path has a high density, and I/O-driven chip-to-chip interconnect technologies are neither necessary nor feasible. Instead, the vertical stacking path is directly driven by a buffer cell within the DRAM and logic chip. The area overhead of the driving circuit is minimal, aligning well with the high-density interconnect characteristics of the vertical stacking path. Unlike the channel analysis method with 50 ohm terminations in Section 2.3, this sub-section combines SeDRAM’s dedicated driving method for cross-process signal integrity analysis.

**Figure 8:**
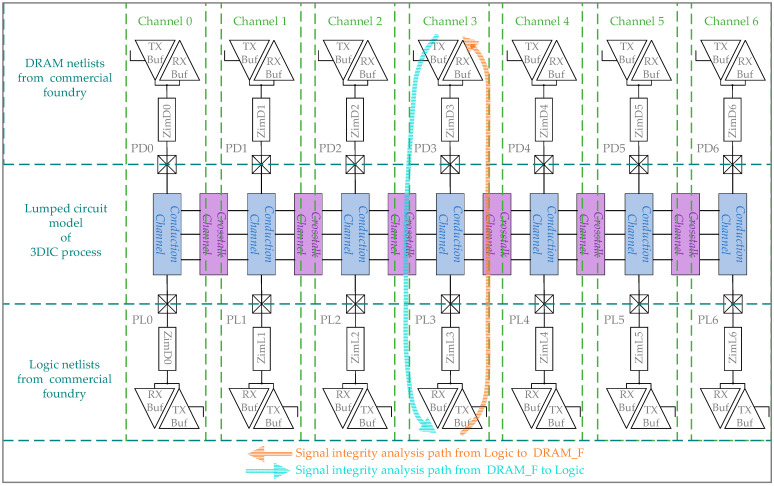
CPSIA frame.

As shown in Figure 8, a CPSIA framework consists of three parts: two kinds of netlists of 25 nm DRAM and 28 nm logic driving buffer based on commercial foundries and the lumped circuit model of the vertical stacking path. The combination of these three simulation elements forms an integrated simulation environment, which includes three processes. This CPSIA environment establishes a 3D SI analysis method equivalent to standard 2D chip design, meeting the requirements of the I/O-less driving structure of SeDRAM and providing greater accuracy than the I/O-based SI analysis. The netlists of DRAM and logic driving buffers include the Transceivers/Receivers (TX/RX) of DRAM_N, DRAM_F, and logic, along with impedances of ZimD0, ZimD1, …, and ZimD6. These impedances represent the inner connecting metal layers between the TX/RX ports and the 3DIC logic interface. The netlists of the DRAM and logic driving buffers include analog behavior described in the DRAM and logic process libraries for signal analysis. The circuit model of the vertical stacking path consists of lumped elements corresponding to mini-TSV and HB cells, enabling cross-process simulations without the need for 3DIC process libraries.

In SeDRAM, all memory access data signals of DRAM_F and DRAM_N are independently designed, with DRAM_F having a longer vertical stacking path, which is the focus of this analysis. In this CPSIA frame, there are two SI analysis paths, as shown in Figure 8. This approach closely approximates the actual circuit’s driver and load responses, avoiding rough evaluation with a 50 ohm driver impedance.

**Figure 9:**
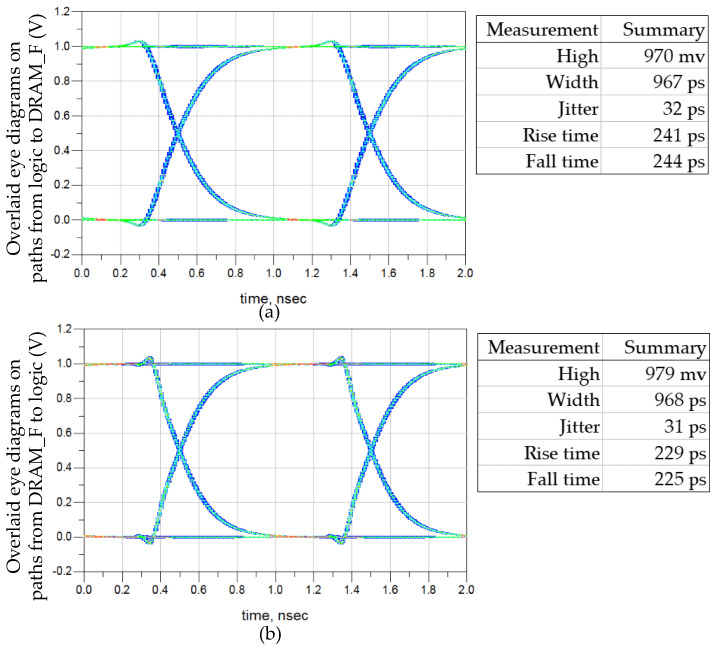
Overlaid eye diagrams at 1 Gbps for the signal integrity analysis. (**a**) Overlaid eye diagrams from logic to DRAM_F. (**b**) Overlaid eye diagrams from DRAM_F to logic.

Pseudo-Random Binary Sequence (PRBS) excitation is applied to seven channels. Due to the synchronous design in the DRAM circuit, eye diagrams of seven channels with the same direction are overlaid, resulting in two sets of eye diagrams. The first set is obtained by overlaying eye diagrams collected with seven logic buffers as the TXs on PD0−PD6, and the second set is obtained by overlaying eye diagrams collected with seven DRAM_F buffers as the TXs on PL0−PL6. The two sets of eye diagrams are shown in Figure 9, corresponding to the two signal integrity paths in Figure 9. The two eye diagrams indicating writing and reading data paths of memory access have high quality and are easily recoverable by RXs, resulting in minimal impacts on DRAM timing. Since the speed of the vertical data paths is below 1 Gbps, the impact of 3DIC is within the tolerance of the vertical stacked DRAM platform.

### 3.2. Impact Analysis Introduced by 3DIC

**Figure 10:**
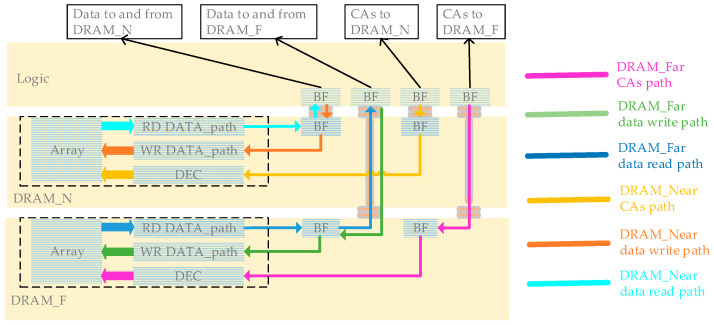
Logic memory access paths.

This sub-section analyzes the impact of 3DIC on memory access. Figure 10 illustrates the Logic Memory Access (LMA) path for logic reading and writing data from and to DRAM_N and DRAM_F. The red, green, and blue lines represent CAs, data writing paths (from the DRAM perspective), and data reading paths of DRAM_F. The yellow, orange, and cyan lines represent CAs, data writing paths (from the DRAM perspective), and data reading paths of DRAM_N. CAs include the command address and tCK, which are unidirectional signals from logic to DRAM, where the address goes through a decoder. Data writing and reading paths connect the DRAM array and interface through the DRAM internal data path. The design and layout of DRAM_F and DRAM_N are identical; in fact, there is only one type of DRAM used in manufacturing the 3DIC wafer, without distinction between stack layers. The design differences in the 3DIC layers enable DRAM_F to extend the data path to the logic interface through the 3DIC structure. The only distinction in the LMA path between DRAM_F and DRAM_N lies in the 3DIC path. The LMA paths of DRAM_N pass through HBs, while the LMA paths of DRAM_F go through a longer 3DIC connection (2HB+1TSV+2HB structure). The degradation of SI in LMA due to 3DIC is evident in terms of signal jitter and signal delay introduced by 3DIC.

**Figure 11:**
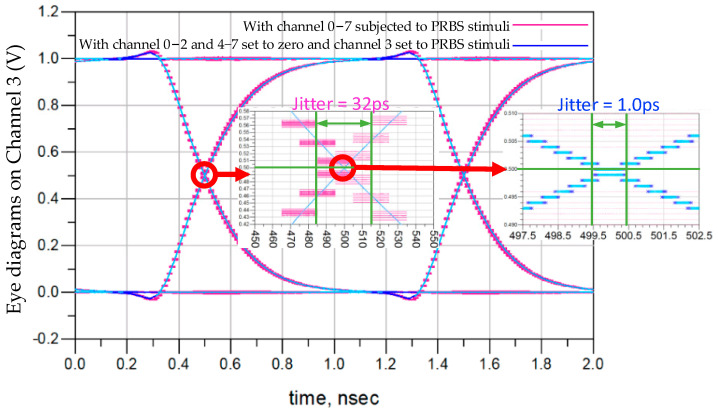
Jitter analysis of data path.

Figure 11 expands on the focus of jitter in the overlaid eye diagrams from logic to DRAM_F. A jitter of 32 ps is observed in the channel response, including a background noise of 1 ps, leading to uncertainty in the sampling time on the DRAM and a reduction in the timing margin for sampling frequency. To isolate the impact of factors other than crosstalk on signal jitter, only the excitation of channel 3 is retained, while the remaining logic TXs are set to zero. A jitter of 1.0 ps is discovered, which is not caused by the flipping of adjacent 3DIC channels. The jitter on the channel response originates from the 3DIC crosstalk channels and is determined by the random encoding of aggressor channels.

**Figure 12:**
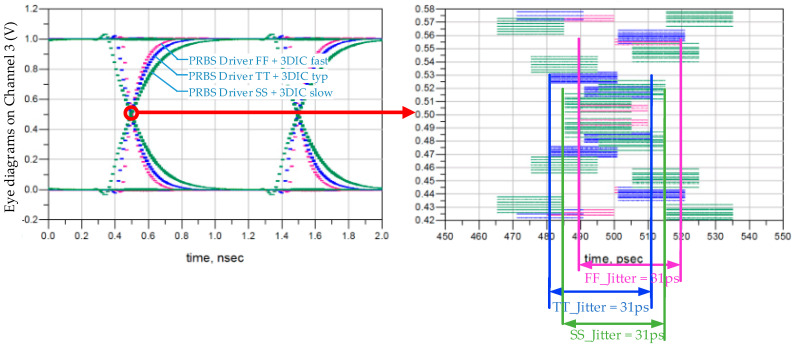
Jitter analysis on data paths with the impact of driver PVT deviation and 3DIC variation.

Figure 12 presents the jitter analysis, considering the impact of driver Process Voltage and Temperature (PVT) deviations as well as 3DIC variation (see Section 2.2). The Driver FF/TT/SS conditions utilize netlists extracted from both the DRAM and logic, featuring the fastest, typical, and slowest combinations of the P-Channel Metal Oxide Semiconductor (PMOS) and the N-Channel Metal Oxide Semiconductor (NMOS). The combination of driver FF and 3DIC fast corresponds to the fastest 3DIC channel, while the combination of driver SS and 3DIC slow corresponds to the slowest 3DIC channel. Figure 12 illustrates the deviations in signal setup time under various conditions. Notably, the absolute values of time-domain jitter remain the same across the three conditions, exhibiting a phase deviation of 4 ps.

The jitter introduced by 3DIC reduces the timing margin of the sampling circuits, becoming a leading cause of the reduced maximum frequency for DRAM_F. Specifically, when writing data from logic to DRAM_F, the jitter on the DRAM data writing path (the green path in Figure 10) decreases the timing margin of the first-level data sampling in the DRAM. When reading data from DRAM_F to logic, the jitter on the DRAM data reading path (the blue path in Figure 10) reduces the timing margin of the first-level data sampling in the logic. The CAs (including clock signals) are driven from logic into DRAM_F through the red path in Figure 10; thus, the jitter on CAs reduces the timing margin of all data sampling in DRAM_F. The 31 ps jitter demonstrated in Figure 11 is a random jitter introduced by crosstalk, which is present not only on the data path but also on the clock net, resulting in a maximum timing uncertainty ranging from 31 ps to 62 ps.

**Figure 13:**
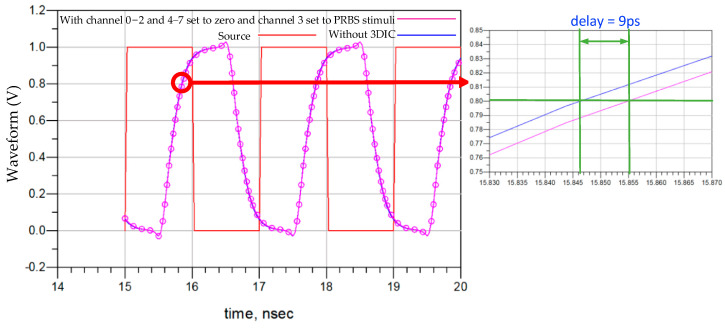
The impact of transmission delay on DRAM_N.

The 3DIC also introduces signal transmission delay. Figure 13 includes the 3DIC driving sources of the buffers, the response of PRBS excitation in the 3DIC channel, and a response without a 3DIC channel between logic and DRAM buffers. There is a 0.7 ns delay between the two responses from the source. In particular, when zooming in on the figure, there is a 9 ps difference between the two buffer responses, using an 80% VDD threshold as the transition from low to high.

The 3DIC load introduces an additional 9 ps delay in the driving response in DRAM_F. The 3DIC delay does not impact writing data to the memory but affects reading data from memory. The data reading circuit belongs to the internal tCK clock domain of DRAM_F, while the sampling data circuit on the logic DIE belongs to the logic tCK clock domain. Only the former undergoes transmission delay introduced by the 3DIC, reducing the timing margin of the first-level sampling in logic.

The jitters introduced by the 3DIC are associated with the behavior of aggressor channels; they are random during the memory access process. Random jitters contribute to the timing uncertainty of DRAM_F memory access, ranging from 31 ps to 62 ps. Furthermore, the delay in the 3DIC path affects the timing of the first-level sampling of DRAM in logic.

### 3.3. Design Optimization of Vertical Stacking

**Figure 14:**
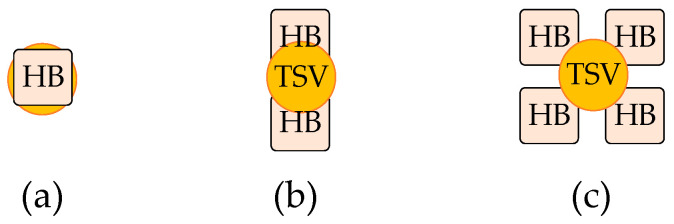
The physical structures of vertical stacking paths: (**a**) the 1HB+1TSV+1HB structure; (**b**) the 2HB+1TSV+2HB structure; (**c**) the 4HB+1TSV+4HB structure.

**Figure 15:**
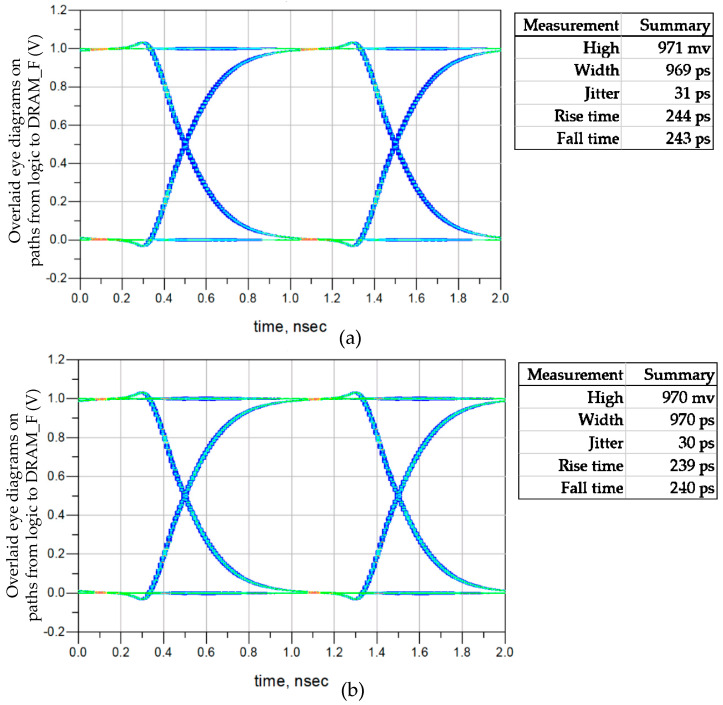
Overlaid eye diagrams from logic to DRAM_F: (**a**) the 1HB+1TSV+1HB structure; (**b**) the 4HB+1TSV+4HB structure.

The diverse combinations of mini-TSV and HB form the vertical stacking path between DRAM_F and logic, as shown in Figure 14. The eye diagrams of the 1HB+1TSV+1HB, 2HB+1TSV+2HB, and 4HB+1TSV+4HB structures are shown separately in Figure 15a, Figure 9a, and Figure 15b. The proportions of HB and TSV cells have a minimal impact on the channel. The performance of these three structures in signal transmission is similar, but they differ in terms of design resource utilization. Mini-TSV cells connect the internal metal layers of the DRAM_N to the HB layer on the backside of the DRAM_N silicon substrate, resulting in an active layer footprint on DRAM_N. Unlike HB cells that do not impact the active layer layout, the number of mini-TSV cells is constrained in DRAM design. The failure ratio of the signal HB is less than 0.1 ppm. In the case of the maximum 64Gb DRAM, there exists a requirement for 300k HB connections carrying critical signals. The employment of the 2HB+1TSV+2HB structure for critical signal connections results in a 3% yield improvement in the 64Gb near-memory product. To prevent the entire DRAM failing due to the bonding failure of a single HB cell, using the 2HB+1TSV+2HB structure to establish the vertical stacking data path represents an excellent tradeoff. Yield is a crucial focus in the large-scale production of SeDRAM. In the collaborative design, a diverse combination of mini-TSV and HB is utilized to create vertical signal and power interconnects.

- A 1HB+1TSV+1HB structure is employed for testing signal interconnects.
- A 2HB+1TSV+2HB structure is employed for interconnecting memory access data signals, such as DQs and CAs. Its advantages include reducing the contact resistance of HB cells in the data path and enhancing the product yield targets.
- A 4HB+1TSV+4HB structure is utilized for the power network. Four sets of HBs in parallel are used to address the high contact resistance issue in HBs, reducing voltage drop and current density in HB cells.

Along with the dedicated buffer driving method, a CPSIA approach is proposed and utilized to analyze the 3DIC jitters, integrating DRAM logic and 3DIC designs in a simulation environment. This approach quantifies the timing uncertainty introduced by 3DIC crosstalk, ranging from 31 ps to 62 ps.

## 4. Physical Testing and Result Analyses

The timing uncertainty, ranging from 31 ps to 62 ps, introduced by the random behavior of aggressor channels coupled through 3DIC crosstalk, was determined. The 3DIC path represents the only difference between DRAM_F and DRAM_N, considering their identical design and layout. Therefore, the quantified 3DIC impact should manifest in the physical testing of SeDRAM. This section provides the physical testing results of the tCK shmoo in a cross-process test structure with commercial DRAM logic and 3DIC manufacturing processes. DRAM_F and DRAM_N from the same 3DIC wafer exhibit an unsymmetric distribution in maximum frequency. Subsequently, a study was conducted to explore the relationship between this phenomenon and the analysis presented in Section 3.

### 4.1. The Test Chip

**Figure 16:**
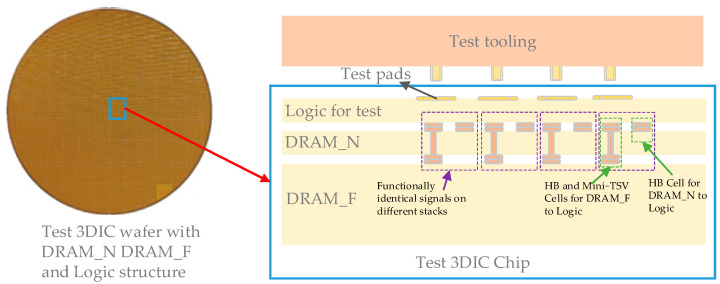
Testing chip environment.

A physical testing wafer is established with a DRAM_N, DRAM_F, and logic stacking structure, as shown in Figure 16. The logic includes DRAM test circuits and test pads used for interconnection with the test tooling. DRAM_N is vertically interconnected with the logic through HB cells; DRAM_F is vertically interconnected with the logic through HB and mini-TSV cells. The vertically interconnected units corresponding to functionally identical signals for DRAM_N and DRAM_F are physically arranged adjacently to reduce channel differences in signals with the same function across the two DRAM stacks.

**Figure 17:**
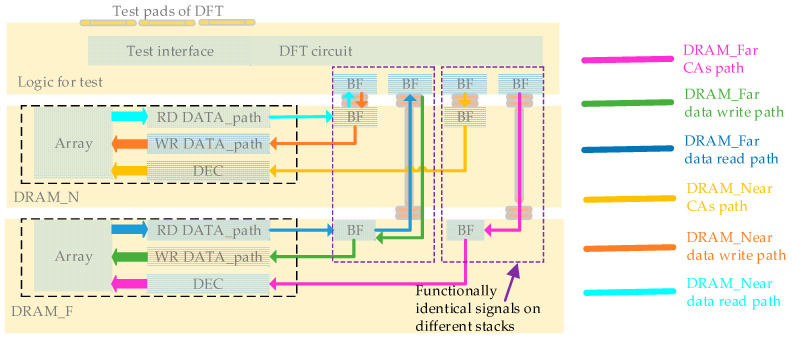
Testing chip structure.

The Logic, DRAM_N, and DRAM_F dies are organized into the testing chip structure through the 3DIC process, as shown in Figure 17. The Logic die includes a Design-for-Test (DFT) circuit used to test the DRAM arrays on DRAM_N and DRAM_F through their LMA interfaces. The memory access path in the test structure is consistent with the memory−compute integration application, including the 3DIC data paths from logic buffers to DRAM_F and DRAM_N buffers. This alignment is also consistent with the cross-process structure shown in Figure 8. The only distinction between the LMA interfaces of DRAM_F and DRAM_N is that the DRAM_F includes a more complex 3DIC path with an HB mini-TSV and HB structure. Under the same 2D chip design, the additional impact of 3DIC on DRAM_F exists in LMA interfaces. This leads to deviations in test results in terms of DRAM_F and DRAM_N.

tCK is the synchronous core clock of the DRAM. The tCK shmoo test follows the standard DRAM testing procedure: a fixed frequency (tCK) is set to perform read and write operations on SeDRAM. Diverse data are written into the SeDRAM, including two DRAM arrays on both DRAM_F and DRAM_N. After reading the operations, the data bus is checked at each Access Time (tAC) step. After scanning through multiple patterns, if all the DRAM arrays pass the write and read loops, the tCK shmoo is marked as a pass (in green); otherwise, it is marked as a fail (in red). The tCK shmoo is a result of extensive scanning of the DRAM arrays with multiple patterns.

**Figure 18:**
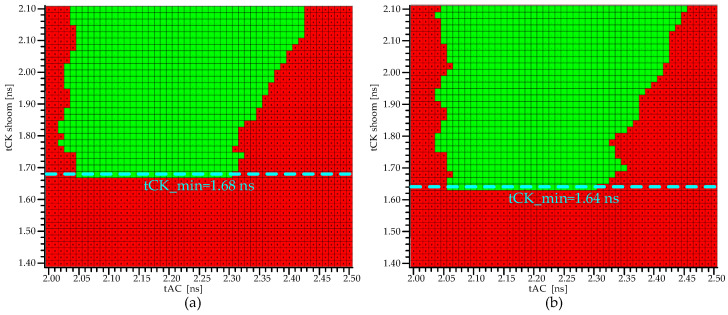
DRAM tCK test shmoo. (**a**) DRAM_F_N. (**b**) DRAM.

Figure 18 shows the tCK shmoo test results of the double-layered DRAM test chip. The shortest tCKs for DRAM_N and DRAM_F are 1.64 ns and 1.68 ns, respectively, with DRAM_F having a slightly lower maximum frequency than DRAM_N. DRAM_F and DRAM_N are two stacked DRAM arrays on the same 3DIC wafer under the same temperature. This tCK Shmoo comparison shows that the minimum tCK (maximum frequency) of DRAM_N is better than that of DRAM_F by 40 ps. The 3DIC is the only distinction between the two DUTs of DRAM_F and DRAM_N. The impact of 3DIC is speculated to be the primary factor influencing this phenomenon.

**Figure 19:**
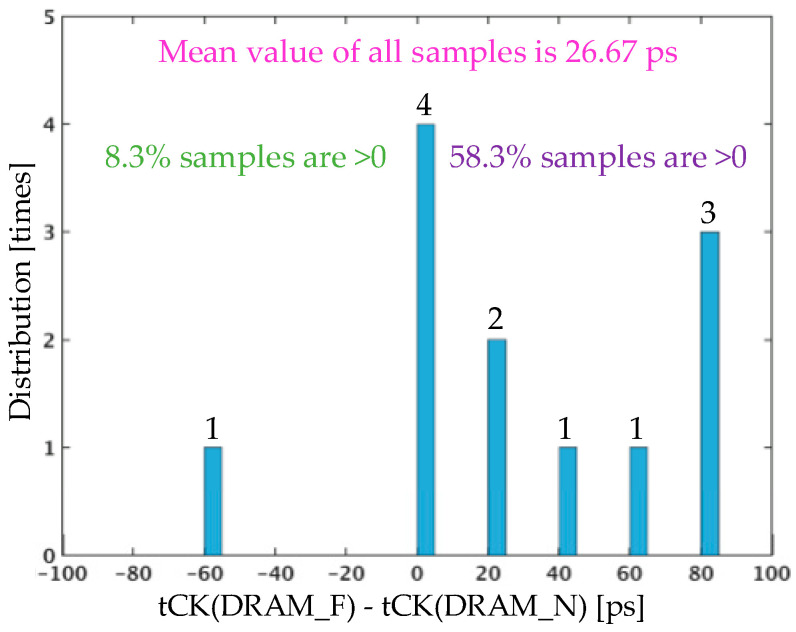
The distribution of tCK_min differences between DRAM_F and DRAM_N.

To illustrate the performance gap between DRAM_N and DRAM_F, 12 sets of samples are depicted in Figure 19. The histogram displays the distribution of the minimum tCK differences between DRAM_F and DRAM_N for the 12 sets of samples. The test results include manufacturing deviations and testing errors, indicating that DRAM_N has a speed advantage over DRAM_F. This is reflected in two aspects: a predominance of positive values over negative values in the distribution of the difference between the tCK_min of DRAM_F and the tCK_min of DRAM_N and the average tCK_min differences, which indicate that the average tCK_min of DRAM_F is smaller than that of the tCK_min of DRAM_N by 26.67 ps. The impact of 3DIC is speculated to be the primary factor influencing this phenomenon.

### 4.2. Analysis of Test Results

The cross-process jitter analysis in Figure 11 shows that 3DIC crosstalk contributes to 31 ps of jitter on the LMA path, with the timing uncertainty of 31 ps introduced by the random behavior of aggressor channels coupled through 3DIC crosstalk, which is the uncertainty in both data and tCK. In the testing environment, the memory access path in DRAM_F, which includes the vertical stacking path formed by HB mini-TSV and HB cells, is distinct from DRAM_N. The tCK path uncertainty of 31 ps reduces the timing margin for all DFF/latch samplings within the DRAM, while the data path uncertainty of 31 ps reduces the timing margin for the first-level sampling in the writing path and the last-level sampling in the reading path. Therefore, this study attributes the 31–62 ps tCK period deviation observed in the tCK shmoo test results to the impact of 3DIC crosstalk.

### 4.3. Model Extension

While the impact of 3DIC on signal integrity meets the requirement of the analysis target below 1 Gbps, this method plays a significant role in determining the evolutionary path of this vertical stacked DRAM platform, reflected in the expansion of stacking structures and the enhancement of LMA speed.

**Figure 20:**
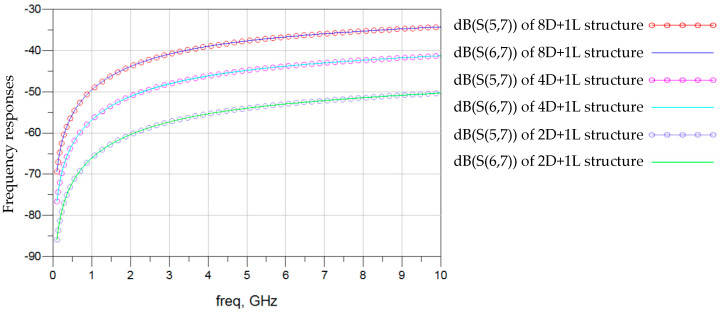
Near-end and far-end crosstalk on termination 8 in the 2D+1L 4D+1L and 8D+1L structures.

The combination of HB and mini-TSV enables us to design higher-stacked DRAM platforms, thereby increasing DRAM density. Lumped circuit models of an eight DRAM and one logic stacking structure and of a four DRAM and one logic stacking structure were established for comparison with the two DRAM and one logic stacking structure analyzed in this paper. Following the frequency analysis method of Figure 4, it was assumed that channel 3 was the victim and channel 2 was the aggressor. The near-end and far-end crosstalk responses of term 5 and term 6 of channel 2 to term 7 of channel 3 are shown in Figure 20. As the stacking structure becomes more complex, the crosstalk introduced by 3DIC gradually increases.

**Figure 21:**
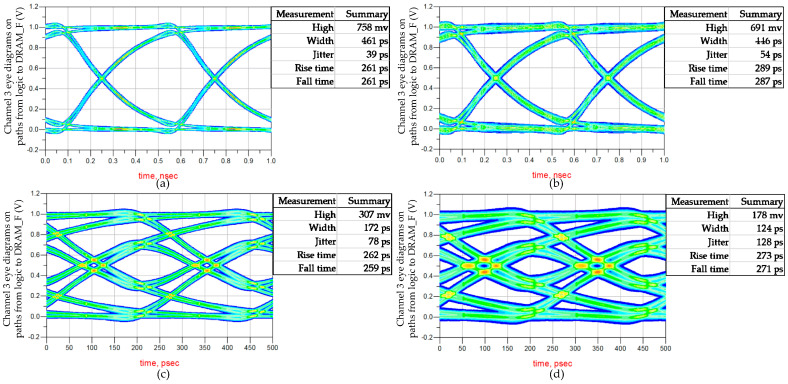
Overlaid eye diagrams from logic to DRAM_F: (**a**) the 2 Gbps on 4D+1L structure; (**b**) the 2 Gbps 8D+1L structure; (**c**) the 4 Gbps on 4D+1L structure; (**d**) the 4 Gbps 8D+1L structure.

According to the objective of this study, the DRAM to logic interface frequency matches the internal clock of the DRAM array. On the roadmap of SeDRAM, we will employ prefetching techniques to fetch data at 8 or 16 times the DRAM array frequency to the DRAM to logic interface, thus enabling data to flow through the DRAM to logic interface at 8 or 16 times the DRAM array frequency. Figure 21 illustrates the eye diagrams for a frequency increase to 2 Gbps and 4 Gbps in stacking structures of 4D+1L (four DRAM layers and one logic layer) and 8D+1L. Above 2 Gbps, the eye diagrams gradually degrade. Combining frequency-domain analysis, the primary cause is return loss. In this next-level structure, the impact of 3DIC cannot be ignored, and quantitative analysis and optimization using this method are necessary.

Based on the relationship between the lumped circuit model and the corresponding physical structure, it is easy to identify three quantitative optimization methods for 3DIC SI, aiming to meet the advancement of SeDRAM, in terms of the expansion of stacking structures and the enhancement of LMA speed. One approach is to increase the pitch of the vertical stacking paths. Another is to introduce direct current channels in the signal vertical stacking path array. The third method focuses on optimizing the 3DIC process through the analysis of key factors leading to 3DIC responses, including structural and material enhancements, such as $C_{\mathrm{TSV}}$ sensitive to the insulation layer thickness.

The jitter analysis result provides an explanation for this interesting physical testing phenomenon, demonstrating the effectiveness of this CPSIA method. The model extension analysis for higher speeds and increased stacking structures illustrates that this method will play a crucial role in SeDRAM’s technological advancements as channel degradation progresses.

## 5. Conclusions

This paper highlights the distinct nature of WoW 3D multi-layer vertical stacked DRAM Platform SI analysis in terms of cross-process and the absence of process segmentation by I/O circuit. A lumped circuit based on the 3DIC physical structure is introduced to establish a modeling methodology for the vertical stacked DRAM platform. All values of the lumped elements in the circuit model are calculated with the transmission line model. In combination with the dedicated buffer driving method, the CPSIA method is proposed and used for the analysis of 3DIC jitters, integrating DRAM logic and 3DIC designs in a simulation environment, determining the timing uncertainty introduced by 3DIC crosstalk ranging from 31 ps to 62 ps. The silicon results show that the distribution of DRAM_N’s maximum frequency is better than that of DRAM_F, with the average of the tCK_min differences being 26.67 ps, demonstrating the effectiveness of this CPSIA method.

## Figures and Tables

**Table 1:** Comparison of cross-process structures.

	HBM [14,15]	Wireless Stacked SRAM [16]	SeDRAM [17,18]
Integration	2.5D	3D	3D
Memory	DRAM	High-density SRAM	DRAM
Stacked Structure	4~8 memory + 1 logic	4 memory + 1 logic	2~8 memory + 1 logic
Vertical Interconnection	TSV + microbump + interposer	None	HB + mini-TSV
	Metal	Wireless	Metal
	Package	None	WoW BEOL
Related Processes	DRAM and logic	Logic 1 and logic 2	DRAM, logic, and 3DIC
Interface between Stacked Memory and Logic	I/O	Coil on logic die	Buffer
Separation of Processes	Yes, by I/O circuit	Yes, by magnetic field	No
SI Analysis Method	Package-based validation	Virtual model	CPSIA

**Table 2:** Physical dimensions of the model indicated in Figure 3a.

	Dimensions	Value	Unit	Description
TSV	$h_{\mathrm{TSV}}$	10	μm	TSV height
	$d_{\mathrm{TSV}}$	2.5	μm	TSV diameter
	$P_{\mathrm{TSV}}$	6	μm	TSV pitch
HB	$h_{\mathrm{HB}}$	2	μm	HB height
	$d_{\mathrm{HB}U}$	0.6	μm	Up part of HB diameter
	$d_{\mathrm{HB}D}$	1.5	μm	Down part of HB diameter
	$P_{\mathrm{HB}}$	3	μm	HB pitch
Insulation Layer	$t_{\mathrm{IL}}$	0.2	μm	Insulation layer thick
IMD1	$h_{\mathrm{IMD}1}$	3	μm	IMD1 height

**Table 3:** Material parameters of the model indicated in Figure 3a.

	Parameters	Value	Unit	Description
TSV	$\rho_{\mathrm{TSV}}$	$1.68\times 10^{-8}$	Ω·m	Resistivity of TSV (Cu)
	$\mu_{r\_\mathrm{TSV}}$	1	\	Relative permeability of TSV
HB	$\rho_{\mathrm{HB}}$	$1.68\times 10^{-8}$	Ω·m	Resistivity of HB (Cu)
Si Sub	$\sigma_{\mathrm{Sub}}$	10	S/m	Conductivity of Si Sub
	$\varepsilon_{r\_\mathrm{Sub}}$	11.9	\	Relative permittivity of Si Sub
IMD1/IMD2	$\varepsilon_{r\_\mathrm{IMD}}$	4.1	\	Relative permittivity of IMD
Insulation Layer	$\varepsilon_{r\_\mathrm{IL}}$	4.1	\	Relative permittivity of insulator
Free Space	$\varepsilon_{0}$	$8.854\times 10^{-12}$	F/m	Permittivity of free space
	$\mu_{0}$	$1.257\times 10^{-6}$	H/m	Permeability of free space

**Table 4:** Lumped element values of the model indicated in Figure 3b.

Function	Medium	Parameters	Value	Unit	Description
Conduction	TSV	$R_{\mathrm{TSV}}$	34.22	mΩ	Equivalent resistance of mini-TSV
		$L_{\mathrm{TSV}}$	3.138	pH	Equivalent inductance of the dual HB structure
	HB	$R_{\mathrm{HB}}$	34.46	mΩ	Equivalent resistance of the dual HB structure
	Insulation Layer	$C_{\mathrm{TSV}}$	2.689	fF	Distributed capacitance between the mini-TSV copper pillar and the DRAM_N substrate
Crosstalk	IMD2	$C_{\mathrm{HB}}$	0.136	fF	Distributed capacitance between adjacent dual HBs
	IMD1	$C_{\mathrm{IMD}}$	0.225	fF	Distributed capacitance between adjacent mini-TSVs
	Si Sub	$G_{\mathrm{Sub}}$	0.144	mS	Equivalent conductance of SI Sub
		$C_{\mathrm{Sub}}$	1.522	fF	Equivalent capacitance of SI Sub

## Data Availability

Simulation and measurement data are available on request from the corresponding author.
